# What are the experiences of colorectal cancer patients with biomarker testing in Canada?: a mixed methods study

**DOI:** 10.1186/s12885-024-12805-6

**Published:** 2024-08-31

**Authors:** Elijah Tongol, Preet Kang, Vicki Cheng, Louise Gastonguay, Felix E. G. Beaudry, Filomena Servidio-Italiano, Mary A. De Vera

**Affiliations:** 1 Faculty of Pharmaceutical Sciences, University of British Columbia, 2405 Wesbrook Mall, Vancouver, BC V6T 1Z3 Canada; 2 Collaboration for Outcomes Research and Evaluation, Vancouver, BC Canada; 3 Clinical Translation, Ontario Institute for Cancer Research, Toronto, ON Canada; 4 Colorectal Cancer Resource & Action Network (CCRAN), Toronto, ON Canada; 5 Centre for Health Evaluation and Outcome Sciences, Vancouver, Canada

**Keywords:** Colorectal cancer, Biomarkers

## Abstract

**Objective:**

Molecular or biomarker testing to guide targeted treatments for colorectal cancer (CRC) has advanced care, specifically by improving treatment specificity. Our objective was to explore patients’ experiences and perspectives with biomarker testing in Canada.

**Methods:**

We conducted a mixed-methods study among adults (≥ 18 years) who have been diagnosed with CRC and able to communicate in English. Quantitative data was gathered using an online survey, with questions on awareness of and experiences with biomarker testing. Qualitative data was gathered using semi-structured interviews with a sample of survey respondents to provide context to survey findings.

**Results:**

Among 55 survey respondents, 76% have heard of biomarker testing and of these, 67% have had biomarker testing done. Among the 33% of respondents that have not had biomarker testing done, reasons were: not offered/referred, fear/anxiety over results, and cost. Respondents who had biomarker testing largely found biomarker testing useful (89%), though, only half indicated that they were able to understand the information on their biomarker testing report. Qualitative analysis of interview transcripts identified four themes: 1) perceived benefits of biomarker testing, 2) knowledge of biomarker testing, 3) experiences with accessing and receiving biomarker testing, and 4) recommendations for addressing challenges with biomarker testing.

**Conclusion:**

Altogether, our study provides insight into CRC patients’ perspectives and experiences with biomarker testing. Ongoing efforts by patient organizations, providers, and policymakers to improve awareness and access to biomarker testing must be informed by the patient perspective.

**Supplementary Information:**

The online version contains supplementary material available at 10.1186/s12885-024-12805-6.

## Introduction

Colorectal cancer (CRC), characterized by the malignant growth of tumors in the colon or rectum, ranks as the third most common malignancy in both males and females in Canada [1] Therapeutic approaches for individuals diagnosed with CRC involve surgery, radiation and chemotherapy [2–4], with palliative therapies also used to manage symptoms and increase survival time for patients with metastatic or stage IV CRC [5]. According to the Canadian Cancer Society in 2023, 5 year-net survival rates for colon cancer was 92% for stage I, 88% for stage II, 68% for stage III, and 11% for stage IV. For rectal cancer, the 5-year survival rates remain at 91% for stage I, 79% for stage II, 74% for stage III and 13% for stage IV [6]. These suggest the prognosis for stage IV or metastatic CRC remains poor and there is an imperative need to improve management.

Biomarkers (or “biological markers” or “molecular markers”) refer to molecules (such as DNA, proteins) found in tumours or other tissues that may reveal normal or abnormal processes in the body [7]. In metastatic CRC, biomarker-informed treatments have shown to significantly improve progression-free and overall survival [8]. CRC biomarkers are used for diagnostic and prognostic purposes, to match patients with more specific treatments, as well as for predictive purposes, that is, with respect to potential responses to treatment options, specifically precision medicines [8]. Indeed, between 2000 and 2023, approximately half of treatments for CRC were approved with associated biomarkers by the United States Food and Drug Administration [9]. In 2017, the American Society for Clinical Pathology, College of American Pathologists, Association for Molecular Pathology, and American Society of Clinical Oncology established guidelines for biomarker testing for epidermal growth factor receptor (EGFR) therapies [10]. In 2022, Canadian consensus practice guidelines for biomarker testing for metastatic CRC were published including minimum recommended testing for KRAS/NRAS, BRAF, and MMR/MSI before starting first-line therapy [11]. The significant impact of biomarker status in CRC outcomes has led to provincial health care reimbursement for automatic (reflex) testing for biomarkers associated with inherited cancer predisposition (specifically Lynch Syndrome) in all patients with invasive CRC across most Canadian provinces [12, 13].

However, biomarker testing uptake is inconsistent and not standard of care across Canadian provinces. Furthermore, evidence suggests a gap in awareness of biomarker testing among patients with cancer, particularly those diagnosed with CRC. In 2021, Colorectal Cancer Canada conducted a pan-Canadian and pan-tumour survey among patients diagnosed with cancer and caregivers. Among 128 respondents, 46.9% indicated having CRC. Nearly half (46.8%) of respondents indicated being unfamiliar with the term “biomarker” and only 26.6% received biomarker testing [14]. In November 2021, the Colorectal Cancer Resource & Action Network (CCRAN) launched *My CRC Consultant*, an evidence-based, biomarker-informed online tool designed for the metastatic CRC patient or their caregiver to encourage informed and joint decision-making between the patient and their treating oncologist. Patients are guided through a series of 13 simple questions requiring their pathology or genomic profiling report; at the end, they receive a personalized report that outlines potential treatment options that may be appropriate for them based on their tumour’s biomarker status. Metrics from *My CRC Consultant* presented at the June 2023 CCRAN Biomarkers Conference revealed that among 236 individuals who accessed the tool in its first year, up to 40% were not aware of their tumour’s biomarker status [15]. Taken together Colorectal Cancer Canada survey findings and metrics from *My CRC Consultant* suggests a knowledge gap on biomarker testing among patients with CRC. Indeed, despite the established clinical utility biomarkers knowledge gaps shown in these previous surveys – including cancer patients being unfamiliar with the term “biomarker” or not knowing aware of their tumour’s biomarker status—are problematic. Indeed, patients’ knowledge of their disease as well as interventions and treatments has been shown to facilitate shared-decision making and adherence and enhancing wellbeing [16]. To better understand these knowledge gaps as well as other issues such as how patients learn about or access biomarker testing and how they perceive and use results, we conducted an explanatory mixed methods study to explore CRC patients’ experiences and perspectives with biomarker testing in Canada.

## Methods

### Study design and participant recruitment

We conducted an explanatory QUAN-QUAL mixed-methods study. This merger of quantitative and qualitative research provides a more comprehensive view and generates more nuanced knowledge and understanding [17]. Specifically, we first conducted a quantitative phase using a survey which then informed recruitment for subsequent qualitative phase which involved interviews, which also explained survey findings.

Inclusion criteria, which we applied to both quantitative and qualitative phases, were: adults 18 years or older, diagnosed with CRC in the last 5 years, and able to read, write, and verbally converse in English. The rationale for limiting to patients diagnosed with CRC in the last 5 years is to capture contemporary experiences with biomarkers following publication of guidelines from the American Society for Clinical Pathology, College of American Pathologists, Association for Molecular Pathology, and American Society of Clinical Oncology [10]. To recruit participants, we advertised the study at The Colorectal Cancer Resource & Action Network (CCRAN) biomarker conference held on June 21–22, 2023. We also used authors’ and affiliations’ social media channels (e.g. Facebook, Instagram, and Twitter) along with a list of individuals who have both previously participated in CRC-related research conducted by our research team and consented to be contacted of future studies. Advertisements included a link to a study website with information on study objectives, eligibility criteria, details of participation, and consent form. Individuals who wished to participate and provided consent were then directed to the study survey; individuals who did not wish to participate were asked to close their browser.

### Quantitative data

Quantitative data was collected using a cross-sectional survey designed using the online platform Qualtrics. Accordingly, this involved a convenience sampling strategy. The survey consisted of five sections with question formats including multiple choice, textbox entry and drop-down responses (Supplementary Fig. 1 provides an overview of the survey). The first section comprised 8 questions on CRC characteristics including age at diagnosis, type (e.g. ‘colon’, ‘rectal’, ‘both sites’), stage (e.g. current diagnosis/treatment), status (e.g. ‘currently undergoing treatment’, ‘completed treatment’), and type of treatments received (e.g. ‘surgery’, ‘radiation’, ‘chemotherapy’). The second section comprised of sequential questions on knowledge and experiences with biomarker testing. Participants may respond to up to 16 questions if they have knowledge of and have had biomarker testing. These include questions on how they heard about biomarker testing (e.g. ‘family physician, ‘patient advocacy group’), how they obtained biomarker testing (e.g. ‘paid myself’, ‘doctor’s referral’), who facilitated access to biomarker testing (e.g. ‘family physician, ‘surgeon’), whether treatment was influenced by biomarker testing, whether there was an actionable mutation/alteration identified, whether they found the biomarker testing useful, whether they had help explaining the meaning of their biomarker testing results, who/what helped explain the meaning of their biomarker testing results (e.g. ‘family physician’, ‘patient advocacy group’), and whether they understood the results and recommendations based on the explanation they received on their biomarker testing results. Given the implications of biomarker testing for supporting shared-decision making, also included in the survey was a third section with 9 questions on participants’ preferences and experiences with shared-decision making through their CRC diagnosis and treatment. The fourth and final section comprised 9 questions on participants’ demographic characteristics including age, sex (e.g. female, male), gender identity (e.g. man, non-binary), race (e.g. South Asian, White, Indigenous), highest level of education (e.g. ‘elementary’, ‘attended some college’), and current household income (e.g. ‘$30,000 and under’, ‘$90,000 to $120,000’). In this section, participants were also asked whether they would be willing to participate in a follow-up one-on-one interview if invited.

Survey data were analyzed using descriptive statistics, including counts and proportions. We also used data visualization methods (e.g., charts, plots). To support quantitative analyses, we used Microsoft Excel 2016, Qualtrics XM Stats iQ, and SPSS. A copy of the survey is provided in Supplementary Material 1.

### Qualitative data

Qualitative data was collected using one-on-one semi-structured interviews with a subset of survey respondents. Sampling was informed by the survey responses as we purposively invited participants based on their responses to the question of whether they have heard of biomarker testing or not. That is, we ensured that we invited both respondents who have heard of biomarker testing and those who have not for interviews. This strategy was key to our mixed methods study design as it allowed us to capture the experiences of survey respondents from both groups. Notably, the sample size for the qualitative study was pragmatically influenced by the availability and willingness of participants, particularly when recruiting and collecting data from individuals who had not heard of biomarker testing. Interviews were conducted by team member (PK) who has experience in collecting qualitative data including interviews and focus groups. Interviews were supported by a topic guide with probes that invited participants to provide greater detail and context for their survey responses. Interviews ranged from 19 to 48 min and were conducted via the Zoom video-conference platform. Following, interviews were transcribed using a professional online transcription software, Sonix [18].

Interview transcripts were analyzed using reflexive thematic analysis. As described by Braun and Clarke [19, 20], reflexive thematic analysis provides an interpretation of the collected data, while recognizing the influence of the researchers’ subjective perspectives. Interview data was analyzed inductively, where the coding and theme development were directed by the content of the collected data [19, 20]. Specifically, our analysis involved the following steps [20]: familiarizing with the data, generating initial codes by systematically reviewing the interviews and coding interesting features of the data. Next, we searched for themes across the codes, reviewing and refining the themes, defining and naming the themes, and finally, reporting the themes. The final sample size for the interviews was determined through an ongoing iterative process; we continuously analyzed the data, which guided further data collection, and engaged in this process until thematic saturation was achieved [21, 22]. Thematic saturation was defined and operationalized in our study by adopting the concept of “theoretical sufficiency” as suggested by Dey and further elaborated by Braun & Clarke [22]. This approach focuses on achieving a sufficient or adequate depth of understanding to build a theory or generate relevant themes and highlights the importance of the quality of data collected – the richness and diversity – over merely the quantity of data. In line with these perspectives, we aimed for data and thematic adequacy, ensuring that our data collection process and themes generated captured a diverse range of experiences and perspectives. As such, in our paper, we used the term “thematic saturation” to reflect the widely used conceptualizations of saturation as information redundancy [22]. To support the qualitative analysis, we used NVivo (International Q. NVivo. 2022).

### Ethical approval and consent

We obtained ethical approval from the University of British Columbia Behavioural Research Ethics Board. Quantitative data were collected and stored securely using Qualtrics which is compliant with the British Columbia (BC) Freedom of Information and Protection of Privacy Act and meeting institutional and jurisdictional privacy requirements. Qualitative data were stored in University of British Columbia servers. Participants provided informed consent prior to participating in the survey and their confidentiality was maintained throughout the study. Specifically, identifiers (i.e., names, email addresses) were gathered and stored separately from survey and interview data and were not used in any outputs related to the study (e.g., manuscript).

## Results

### Quantitative results

Altogether, 55 patients with CRC completed the survey between 21/06/2023 and 24/08/23. Table 1 summarizes the participants’ demographic and CRC characteristics according to those who had heard of biomarker testing (*n* = 42, 76%) and those who had not heard of biomarker testing (*n* = 13, 24%).

**Table 1:** Participants’ Demographic and CRC Characteristics (*n* = 55)

	**Have heard of biomarker testing** **(*n* = 42)**	**Have not heard of biomarker testing** **(*n* = 13)**
**Demographic characteristics**		
Age (mean, SD)	47.8 ± 13.5	47.9 ± 12.3
**Sex (*n*, %)**^**a**^		
Female	29 (70.7%)	7 (63.6%)
Male	12 (29.3%)	4 (36.4%)
Level of education (*n*, %)*		
Secondary school	1 (2.4%)	1 (9.1%)
Some college and/or university	4 (9.8%)	2 (18.2%)
Graduated 2-year college, technical school, and/or university	3 (7.3%)	1 (9.1%)
Graduated 4-year college, technical school, and/or university	19 (46.3%)	4 (36.4%)
Post-graduate degree	14 (34.1%)	3 (27.3%)
**Canadian province/territory of residence (** *n*, %)*		
Ontario	25 (55.6%)	5 (55.6%)
British Columbia	12 (26.7%)	3 (33.3%)
Nova Scotia	2 (4.4%)	1 (11.1%)
Alberta	2 (5.6%)	
Manitoba	1 (2.2%)	
Newfoundland and Labrador	1 (2.2%)	
Prince Edward Island	1 (2.2%)	
Saskatchewan	1 (2.2%)	
**CRC Characteristics**		
** Age at diagnosis (** *n*, %)*		
Early age onset CRC (< 50 years)	24 (57.1%)	7 (63.6%)
Average age onset CRC (≥ 50 years)	18 (42.9%)	4 (36.4%)
**Cancer stage at diagnosis (** ***n*, %)**		
Stage I	4 (9.5%)	2 (15.4%)
Stage II	9 (21.4%)	1 (7.7%)
Stage III	13 (31%)	6 (46.2%)
Stage IV	16 (38.1%)	3 (23.1%)
Don’t know	0	1 (7.7%)
**Cancer site at diagnosis (*n*, %)**		
Colon	30 (71.4%)	7 (53.8%)
Rectum	11 (26.2%)	4 (30.8%)
Both	1 (2.4%)	2 (15.4%)
**Current diagnosis/treatment status (** ***n*, %)**		
Newly diagnosed (have not started treatment)	2 (4.8%)	0
Currently in treatment	17 (40.5%)	3 (23.1%)
Completed treatment	23 (54.8%)	10 (76.9%)

^a^Proportions calculated based on completed responses (some have missing data)

**Fig. 1:**
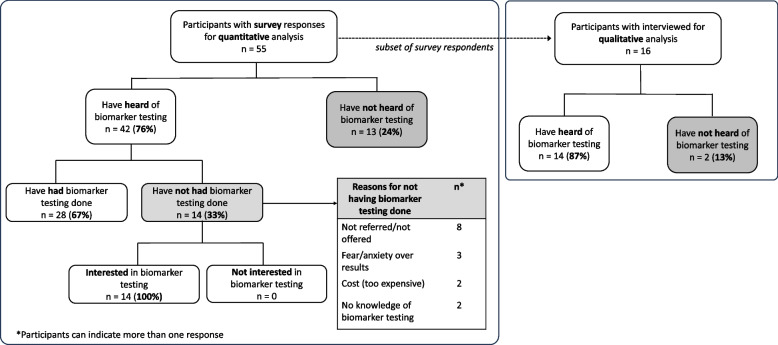
Flow of participants for survey (quantitative, *n* = 55) and semi-structured interviews (qualitative, *n* = 16)

As shown in Fig. 1 among 42 survey respondents that have heard of biomarker testing, 67% had undergone tests and 33% have not. Reasons for not undergoing biomarker testing include not being referred/not offered tests (47%), fear or anxiety over results (18%), and cost (12%).

**Fig. 2:**
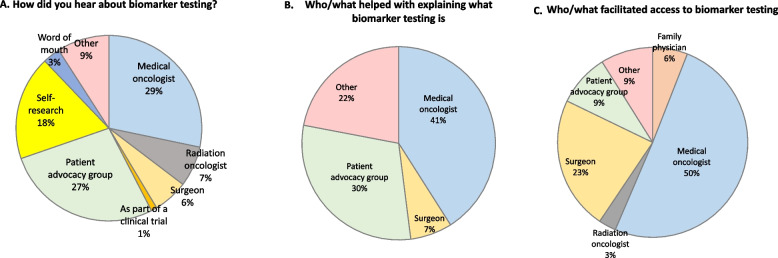
Experiences with awareness, knowledge, and access to biomarker testing (*n* = 42 participants)

Survey responses that quantify experiences with biomarker testing are visualized in Fig. 2. Medical oncologists played the most prominent role in terms of raising awareness (“how did you hear about biomarker testing”) and educating (“who/what helped with explaining the meaning of biomarker testing”) about biomarker testing and facilitating access. Patient advocacy groups also played a role in raising awareness and educating about biomarker testing.

**Fig. 3:**
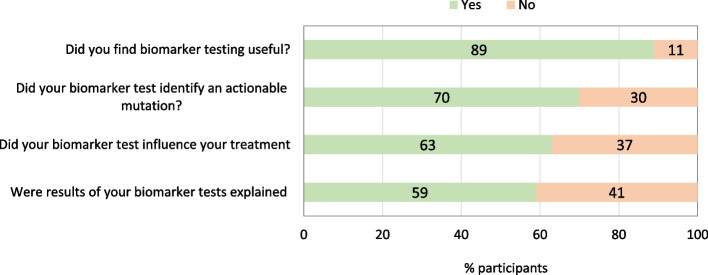
Experiences and perspectives following biomarker testing (*n* = 28 participants)

Figure 3 shows experiences and perspectives following biomarker testing. The majority (89%) indicated that they found biomarker testing useful, 70% indicated that tests identified actionable mutation(s), and 63% indicated that tests influenced treatment. However, just over half (59%) indicated that results of the biomarker tests were explained.

**Fig. 4:**
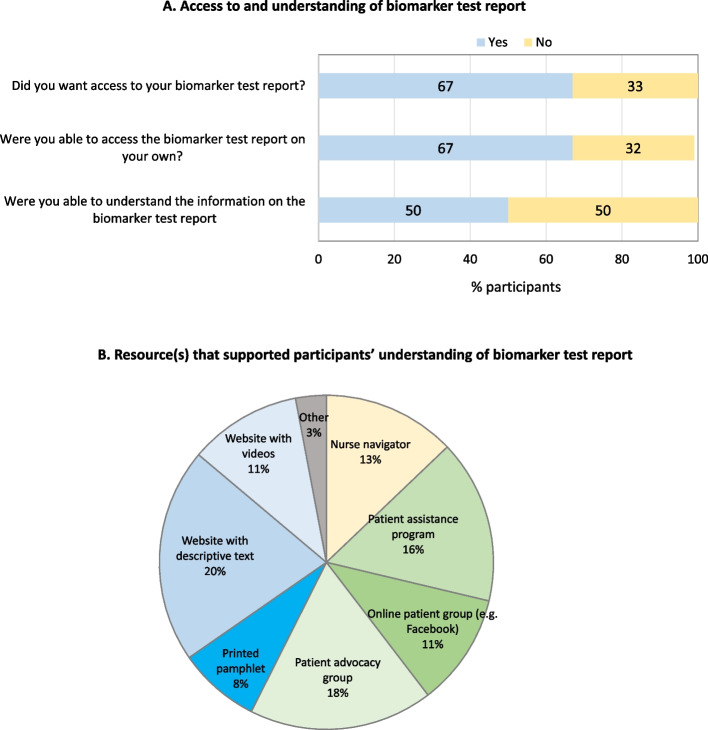
Experiences and perspectives on biomarker test report (*n* = 28 participants)

Figure 4 summarizes experiences with biomarker test reports. Two-thirds (67%) of participants indicated that they were able to access their biomarker test report, however, just half (50%) shared that they were able to understand the information on the report. When further queried on what resource(s) helped facilitate understanding of their biomarker test report, responses suggested the utility of information-based resources, namely websites either with descriptive text (20%) or video (11%) as well as patient-support resources, including patient advocacy group (18%), patient assistance program(s) offered by pharmaceutical/biotechnology companies (16%), and online patient groups such as those on Facebook (11%).

### Qualitative results

From survey respondents, 16 participated in interviews, including 14 who have heard of biomarker testing and 2 who have not heard of biomarker testing (Fig. 1). Thematic analyses of interview transcripts led to the development of four themes. Representative quotes that support the description of these themes are also provided, characterizing participants according to whether they received biomarker testing or not, as a lens for contextualizing findings and supporting interpretations.

The first theme describes participants’ perceptions on the benefits of biomarker testing. Participants who received biomarker testing shared that biomarker testing not only had physical health impacts, that is in helping to inform CRC treatments, they also impacted mental health in terms of *relief* in dealing with their diagnosis, as expressed by a participant, “*even though it didn’t turn out to…give me any actionable information, I'm still glad that I got [biomarker testing]. Like if I was to go back, I would still do it again because of that extra peace of mind that I did everything that I could do*.” (P3, received biomarker testing). Words shared by participants to describe biomarker testing, such as “*very important*” and “*valuable*” reflect CRC patients’ perspectives. Even participants who did not receive biomarker testing shared what they perceived as benefits for them as captured by this quote: “*I think if I’d had biomarker testing done, then it would have been more decisive [about possible treatment]*” (P6, did not receive biomarker testing).

The second theme describes participants’ knowledge of biomarker testing. Apart from one participant who demonstrated in-depth knowledge, participants indicated largely limited knowledge of biomarker testing, despite indicating on the survey that they had heard of biomarker testing. For example, sentiments such as “*I know nothing*” (P15, did not receive biomarker testing) were shared. Even those who received biomarker testing shared that they knew very little as reflected by this quote: “*I don’t know much about biomarker testing*” (P16, received biomarker testing). However, participants did indicate their awareness of resources available for educating patients on biomarker tests. Participants indicated that their oncologists were a primary resource for their knowledge of biomarker tests. The CRC community, including other patients and patient advocacy organizations also played a role as reflected by the following quote that *“a lot of the knowledge around the testing [came] from community members and organizations in the colon cancer space”* (P9, received biomarker testing)*.* Indeed, qualitative interviews provided further context to the role that patient advocacy organizations played: these tended to provide additional (to expand on that provided by oncologists) or clarifying information as captured by this quote from a participant who “*primarily relied on conversations like what my oncologist could tell me… and get in contact with CCRAN [a patient advocacy organization] for more information’”* (P16, received biomarker testing).

The third theme captures participants’ various experiences with accessing and receiving biomarker testing. Among 4 interviewed participants with Stage 4 CRC, 3 received biomarker testing; among 7 interviews participants with Stage 3 CRC, 4 received biomarker testing. In terms of accessing biomarker testing, one participant with Stage 3 CRC pursued this privately by *“paying a lot of money*” (P2, received biomarker testing) out of pocket as their cancer center denied access upon request, while another participant with Stage 3 received access through self-advocacy within the same organization, sharing *“I basically had to fight for a referral to [the cancer center for biomarker testing]*” (P7, received biomarker testing). Participants who did not receive biomarker testing shared various barriers to access including at the health care provider level (was not provided with information on biomarker testing) or organizational level, with one participant sharing that they were told that “*[biomarkers] are not standard of care”* (P16, did not receive biomarker testing). In terms of receiving biomarker testing, participants particularly touched on testing reports, nothing that these contained overwhelming information—“*the [biomarker test] report is a little too complicated… with links to academic journal, which I don’t really understand” (*P2, received biomarker testing). In terms of supports for understanding and interpreting biomarker testing reports, many shared that these were quite limited. Though one participant shared that they appreciated the clarity they received when their oncologist “*went through the report line by line with [them]”* (P18, received biomarker testing).

A fourth theme captured participants’ recommendations for addressing challenges with biomarker testing. With respect to addressing knowledge gaps on biomarker testing, participants shared their desire to receive “*resource pages or a pamphlet that explain more about biomarker testing from cancer centers*” (P12, did not receive biomarker testing) or “*short videos from organizations*” (P14, did not receive biomarker testing) that outlines the importance and benefits of biomarker testing. A practical recommendation would involve presenting “*straightforward [information] with applicable or possible treatment options…[in] a very comprehensive website*”. (P2, received biomarker testing) with “information specific to certain cases” (P8, biomarker testing status unknown). Interviews also with participants also highlighted practical recommendations for addressing challenges with overwhelming and lengthy biomarker testing reports. One participant recommended the development of *“a tool where I could put in the results of the genetic testing and it would be able to let you know there’s no targeted or immunotherapy is available for you or here are some questions you should ask your health care provider”* (P9, received biomarker testing). Another participant suggested that biomarker testing reports could incorporate *“visual or graphic aids”* (P4, biomarker testing status unknown) to supplement the information presented. Additionally, several participants emphasized the importance of having concise and straightforward language in the report itself.

## Discussion

Combining quantitative data from an online survey and qualitative data from semi-structured interviews, we sought to better understand the experiences and perspectives of patients with CRC with biomarker testing. The majority of survey respondents have heard of and undergone biomarker testing. Those that have not had biomarker testing was due to: not being referred/offered tests, fear/anxiety over results, costs, and not knowing about tests. While quantitative findings provide evidence for the utility of biomarker testing (with 89% of respondents indicating that they found these useful), they also suggest areas for improvement particularly with test reports, with only half of respondents indicating that they were able to understand the information. Qualitative findings provide further context – highlighting the perceived impacts of biomarker testing both in terms of physical and mental health, as well as identifying challenges, particularly with accessing biomarker testing. Significantly through this better understanding, our study has implications for informing approaches to improve awareness and access to biomarker testing among patients with CRC.

To our knowledge, this is the first study to combine quantitative and qualitative research approaches to better understand experiences and perspectives of CRC patients towards biomarker testing. Quantitative studies that have addressed similar research questions are limited. A 2021 survey of 128 patients with cancer and caregivers in Canada reported that 46.8% of respondents indicated that they are unfamiliar with the term “biomarker” and 69.1% unaware that “biomarkers can help determine the best treatment when diagnosed” [14]. Among survey respondents, only 26.6% received biomarker testing, suggesting a lack of knowledge and awareness about biomarker testing in Canada, particularly at the patient level. In 2023, Fortune et al. [23] examined patient biomarker testing experiences for 436 patients with cancer in the United States including lung (*n* = 164), breast (*n* = 155), and colorectal (*n* = 117). Their results showed high biomarker testing rates (85%), with patient factors such as health literacy, informational needs, education, and prior experience working in the healthcare field affecting patient’s familiarity with biomarker testing [23]. Despite the high testing rates, the study emphasized the need of reducing informational gaps by improving health literacy and enhancing patient-provider communication to further enhance patient understanding of biomarker testing [22]. While our study focused on characterizing the perspectives and experiences of CRC patients specifically, we also observed moderately high rates of biomarker testing (67%). Moreover, most patients (41%) engaged in discussions with their oncologists regarding the test results, aligning with Fortune et al.'s findings indicating that most patients learned (71%) and had conversations (75%) about biomarker testing with their oncology team [22]. While oncologists seem to play a significant role in shaping patient understanding of biomarker testing, our study also highlights the influence of patient advocacy groups in biomarker testing. This is of particular and practical relevance given that patient advocacy groups may be able to provide resources or informational support or link patients to those who have had similar experiences. Furthermore, patient advocacy groups may also support patients to access biomarker testing.

Qualitative studies are even more scarce with respect to CRC patients’ experiences with biomarker testing and we draw on prior qualitative research with patients with other tumour types. In 2023, Pack et al. conducted interviews exploring the experiences of cancer care providers (*n* = 15) and non-small cell lung cancer patients (*n* = 12) in the United States (US) regarding biomarker testing [24]. While patient participants shared that they had general awareness about what biomarker testing is and what it is used for, they had knowledge gaps with respect to processes involved. Of concern, many patients revealed not knowing of their biomarker testing results. Both provider and patient participants shared the lack of patient-friendly education material on biomarker testing. With respect to biomarker testing results, participants shared that these were mostly received verbally, particularly from their oncologists, although some described reports that were overwhelming (e.g., too much medical and/or scientific jargon) and difficult to understand. The LUNGevity report [25] on experiences of patients with lung cancer in the US with biomarker testing also highlighted challenges with interpreting the results of biomarker testing, as captured in a participant’s quote, *“in my report, …genetic findings, EGFR exon 19 deletion, no problem. But then in parentheses, there’s an E746_T751* > *L, close parentheses. What does that mean? To this day, I have no idea what that in the parentheses mean”*. From the findings of the LUNGevity report, even healthcare providers emphasized that *“it’s impossible for a lay person to follow this. It’s just not reasonable and not possible”*. While participants in our study shared similar concerns about lack of knowledge about biomarkers and challenges in interpreting biomarker testing reports on their own, they additionally offered practical recommendations for addressing these challenges. This highlights the importance of including the patient perspective into the development of materials and tools for educating and supporting patients about biomarker testing.

It is important to discuss limitations of our study. While our study provides insights in the experience of some CRC patients in Canada, online recruitment and quantitative data collection limits our study to individuals with access to the Internet and/or those who engage in online CRC communities and channels. Furthermore, as participants chose whether to complete the survey or not, we do not have any knowledge about those who chose not to access the survey or those who ended the survey before completing. While these limitations may be due to the relatively small sample size of 55 for the quantitative analysis, this may also reflect the larger issue of lack of awareness of biomarker testing among CRC patients overall, particularly in Canada. Essentially, participants who accessed our study may be biased towards those who have knowledge and awareness of biomarker testing. Related to this, limiting our inclusion to English-speaking participants may further bias our results towards those with access and awareness and perpetuate the absence of patients from underrepresented communities. Finally, qualitative findings presented a small sample size of participants who had not heard of biomarker testing, with only two interviews conducted in this group. The availability and willingness of participants to engage in interviews were key factors influencing the sample size of this group, which we acknowledge as a limitation. Future research should aim to recruit a larger and more diverse sample to better capture the experiences and perspectives of individuals unfamiliar with biomarker testing.

It is important to discuss implications of our study, particularly in highlighting the importance of understanding CRC patients’ knowledge about biomarker testing and addressing health information needs. Indeed, cancer patients’ knowledge of their disease as well as interventions and treatments contribute to shared-decision making and adherence and enhances patients’ wellbeing [16]. Cancer patients’ knowledge and understanding of precision medicine is particularly important to continue advancing this area and improving patient outcomes [26]. This is why findings such as ours, consistent with prior findings by Colorectal Cancer Canada [14] and CCRAN [15] that CRC patients are not aware of biomarker testing is problematic as they suggest potential missed opportunities for patients. Our study attempted to address this knowledge gap by identifying how and where CRC patients learn about biomarker testing as potential informational targets. With medical oncologists (29%) and patient advocacy groups (27%) reported as the most common sources by study participants for hearing about biomarker testing, approaches to supporting and/or enhancing these roles are warranted. For example, a call-to-action for providers is the development and promotion of targeted oncology education programs to increase awareness and understanding of biomarker testing [15]. With respect to patient advocacy groups, collaboration to address issues that are common or shared across tumour sites will amplify efforts; there are also calls for developing information and self-advocacy tools [15], and we advocate for a “for patients, by patients” approach. Such recommendations are in line with addressing patients’ health information needs [27], that are consistent with seeking behaviours of those diagnosed with CRC [28]. Finally, it is also important to discuss recommendations for future research on experiences of patients with biomarker testing. To address aforementioned limitations of the current study, considerations of equity, diversity, and inclusion are important. This would include aiming for representative study samples and ensuring that the study is accessible (e.g., survey in other languages). Along with this comes the need for more diverse recruitment strategies – including both online (e.g., patient advocacy organization websites in addition to social media and other platforms used in the present study) and offline (e.g., posters in cancer care centres, hospitals) approaches.

Overall, as the importance of biomarker testing in CRC continues to grow, our study provides insight into patients’ perspectives and experiences. Key findings of the perceived usefulness of biomarker testing among participants who were able to receive the test underlie the significance of continued efforts to support knowledge of biomarker testing and understanding of results for CRC patients.

### Supplementary Information

Supplementary Material 1.

Supplementary Material 2.

## Data Availability

The data underlying this article will be shared on reasonable request to the corresponding author.
