# Supplementary material for: What are the experiences of colorectal cancer patients with biomarker testing in Canada?: a mixed methods study
Source: BMC Cancer. 2024 Aug 31;24:1076. doi: 10.1186/s12885-024-12805-6 (PMC11365144; doi:10.1186/s12885-024-12805-6)
Supplement: Supplementary file 1 — Supplementary Material 1. [file 12885_2024_12805_MOESM1_ESM.docx]

****Recording on Zoom****

Hi! Thank you so much for joining me here today. This discussion will be a one-on-one session and will be approximately 30 to 60 minutes long; a reminder that this interview will be recorded, please start the session with your video turned on so I can verify identifying details and then you can choose to keep your video on or off. ((I will be taking typed notes on my other screen here, so if I’m not facing you directly at times, I’m just typing some notes down but definitely still listening.)) During this interview, I would like to hear about your perspectives, attitudes, and experiences with biomarker testing, as well as understand any facilitators and barriers that you encountered with regards to accessing the test. There will be time at the end of the session for you to ask me questions. If you don’t feel comfortable answering something we can move on, and you should answer the way you feel comfortable. We can provide you with a list of resources if you feel the need .Do you have any questions so far about the purpose of the interview and study?

Any information you provide will be kept confidential. In order to maintain confidentiality during this interview, if you happen to refer someone by name, we will remove this name from our analysis. We may use some quotations without identifying its author in the research publication.

I also have your signed consent form for this interview. As you know, this interview will be audio recorded so we can analyze the results for research purposes only. Do you have any questions before we begin?

- **Okay, I will start the recording/digital recorder NOW.**

1. [ALL PARTICIPANTS]

Could you please start off with sharing your CRC journey and experience with me?

- 1. Where are you now in your CRC journey? (e.g., newly diagnosed, undergoing active treatment, post-treatment?)

1. Have you heard of biomarkers before completing the survey?

(Did you receive biomarker testing since completing of the survey?)

If No

Can you please share with me your knowledge and understanding of biomarker testing so far?

- 1. What resources do you usually use to get medical advice, aside from meetings with your physician/oncologist? (books, articles, online research, others?)
  2. What sort of resource would help you interpret information about biomarkers?
  3. Is there any conferences or seminars about biomarkers (in person or on line) that you attended about your cancer?
  4. What barriers do you think might have prevented you in getting about biomarkers information?
  5. Do you have particular feelings or emotion in regards to biomarkers testing?

(fear, anxiety, stress, lack of confidentiality…)

- 1. ….

1. If you were to receive biomarker testing, would you want to access your report yourself or having your oncologist only to receive it?

No: Why?

Yes: Why? And in what form would you like to receive it (e.g. verbally, online, on paper)?

**If YES**

- 1. Who recommended you receive biomarker testing? (e.g., oncologist referral, ?) *🡪 address facilitation to access*
     1. Who/what facilitated your access to get biomarker testing?
     2. What Type of biomarker testing
     3. Are you familiar with “my CRC consultant” from CCRAN.

 (a patient focus organisation) My Colorectal Cancer Consultant is an online tool designed by CCRAN to provide the advanced colorectal cancer patient with information about potential treatment options they can discuss with their treating oncologist.

- 1. How did you as a patient utilize the results of your biomarker test?
     1. Did your oncologist share with you the meaning of your biomarker test results?
     2. How did your oncologist utilize the results of your biomarker test? (e.g., to guide your treatment?)
     3. As a patient with CRC, do you feel like receiving a biomarker test was beneficial to your overall treatment and care?
  2. In what context were your biomarker results communicated with you (e.g. verbally, online, on paper)?
     1. Did you as a patient handle the biomarker report on your own?

Or did your oncologist tend to summarize the results?

- 1. Would you want access to your report? Why?

In what form (e.g. verbally, online, on paper)?

1. [PARTICIPANTS **WHO RECEIVED** BIOMARKER TEST] This concludes the interview. But before I turn the recorder off, is there anything else about your experience with biomarker testing that is important to you that you would like to share?
2. [PARTICIPANTS WHO **DID NOT RECEIVE** BIOMARKER TEST] This concludes the interview. But before I turn the recorder off, do you have any expectations or aspirations regarding biomarker testing for CRC patients in Canada that you would like to share?
3. [ALL PARTICIPANTS] Do you have any other questions you would like to ask me?

Turn off recording.

**FOR OUR INFORMATION, to share if needed after the interview if ask?**

**What is biomarker testing for cancer treatment?**

Biomarker testing is a way to look for genes, proteins, and other substances (called [biomarkers](https://www.cancer.gov/Common/PopUps/popDefinition.aspx?id=CDR0000045618&version=Patient&language=en) or tumor markers) that can provide information about cancer. Each person’s cancer has a unique pattern of biomarkers. Some biomarkers affect how certain cancer treatments work. Biomarker testing may help you and your doctor choose a cancer treatment for you.

There are also other kinds of biomarkers that can help doctors diagnose and monitor cancer during and after treatment. To learn more, visit the [Tumor Markers fact sheet](https://www.cancer.gov/about-cancer/diagnosis-staging/diagnosis/tumor-markers-fact-sheet).

Biomarker testing is for people who have cancer. People with [solid tumors](https://www.cancer.gov/Common/PopUps/popDefinition.aspx?id=CDR0000045301&version=Patient&language=en) and people with [blood cancer](https://www.cancer.gov/Common/PopUps/popDefinition.aspx?id=CDR0000733834&version=Patient&language=en) can get biomarker testing.

Biomarker testing for cancer treatment may also be called:

- tumor testing
- tumor genetic testing
- genomic testing or genomic profiling
- molecular testing or molecular profiling
- somatic testing
- tumor subtyping

A biomarker test may be called a [companion diagnostic test](https://www.cancer.gov/Common/PopUps/popDefinition.aspx?id=CDR0000797062&version=Patient&language=en) if it is paired with a specific treatment.

Biomarker testing is different from [genetic testing](https://www.cancer.gov/about-cancer/causes-prevention/genetics/genetic-testing-fact-sheet) that is used to find out if someone has [inherited](https://www.cancer.gov/Common/PopUps/popDefinition.aspx?id=CDR0000045098&version=Patient&language=en) mutations that make them more likely to get cancer. Inherited mutations are those you are born with. They are passed on to you by your parents.
