# Supplementary material for: What are the experiences of colorectal cancer patients with biomarker testing in Canada?: a mixed methods study
Source: BMC Cancer. 2024 Aug 31;24:1076. doi: 10.1186/s12885-024-12805-6 (PMC11365144; doi:10.1186/s12885-024-12805-6)
Supplement: Supplementary file 2 — Supplementary Material 2. [file 12885_2024_12805_MOESM2_ESM.docx]

Supplementary Material 1: Quantitative Survey

**Section 1: Questions about experiences with colorectal cancer**

- **At what age were you diagnosed with colorectal cancer?**
- **When were you diagnosed with colorectal cancer?** (MM/YYYY)
- **What type of colorectal cancer were you diagnosed with?**

Colon

Rectal

Both

- **What stage of colorectal cancer were you diagnosed with?**

Stage 0

Stage I

Stage II

Stage III

Stage IV

Do not know

- **What type of doctor gave you your diagnosis?**

Family doctor

Gastroenterologist

Oncologist

Surgeon

Other __________________________________________________

- **What is your current diagnosis/treatment status?**

Newly diagnosed (have not started treatment yet)

Currently undergoing treatment

Completed treatment

- **What type of treatment have you received?** (Please select all that apply)

Surgery

Radiation

Chemotherapy

Immunotherapy

No treatment

Other __________________________________________________

- Have you been diagnosed in the past with another type of cancer?

No

Yes (Please indicate what type and year of diagnosis) __________________________________________________

**Section 2**

**2A: Questions about knowledge and experiences with biomarker testing**
**Have you heard about biomarker testing before?**

No

Yes

- **How did you hear about biomarker testing?** (Please select all that apply)

Family physician

Medical oncologist

Radiation oncologist

Surgeon

As part of a clinical trial

Patient advocacy group

Self-research

Word of mouth

Other __________________________________________________

- **Have you had biomarker testing done?**

No

Yes

- **What are the main reasons you did not have biomarker testing done?** (Please select all that apply)

Concerns about confidentiality

Cost (too expensive)

Do not understand the relevance or importance of biomarker testing

Fear/anxiety over results

Never heard about biomarker testing before

Skepticism (do not believe biomarker testing will be helpful)

Was not referred to/offered biomarker testing

Other __________________________________________________

- **If you had been offered more information about biomarker testing, would you be interested in receiving it?**

No

Yes

**2B: Questions about your experiences with biomarker testing and the results**

- **In which country was your biomarker testing performed?**

Canada

USA

Other __________________________________________________

- **How did you obtain biomarker testing?**

On my own (paid myself)

Through a doctor's referral

Other __________________________________________________

- **Who/what facilitated your access to biomarker testing?** (Please select all that apply)

Family physician

Medical oncologist

Radiation oncologist

Surgeon

As part of a clinical trial

Patient advocacy group

No one/ none facilitated access

Other __________________________________________________

- **Was your treatment for your colorectal cancer influenced by your biomarker testing results?**

No

Yes

- **Did you find your biomarker test results useful?**

No

Yes

- **Was an actionable mutation/alteration identified from your biomarker testing results?**

No

Yes

- **Did you have help with explaining the meaning of your biomarker testing results?**

No

Yes, if Yes: __________________________________________________

- **Who/what helped with explaining the meaning of your biomarker testing results?**

Family physician

Medical oncologist

Radiation oncologist

Surgeon

As part of a clinical trial

Patient advocacy group

Other__________________________________________________

- **Based on the explanation you received, did you feel you understood the results and recommendations from your biomarker testing results?**

No

Yes

**2C. Questions about your experiences with biomarker test report**

- **Were you able to access the biomarker testing report on your own?**

No

Yes

- **In what format(s) were you able to access your biomarker testing report?** (Please select all that apply)

A printed hand-out

Online

Other __________________________________________________

- **Based on the information in the biomarker testing report, did you feel you understood the results and recommendations?**

No

Yes

- **What resource(s) would help you understand information on your biomarker testing report?** (Please check one or more responses)

Biotech company patient assistance program

Nurse navigator

Online patient group (e.g., Facebook)

Patient advocacy group

Pharmaceutical company patient assistance program

Printed pamphlet

Website with descriptive text

Website with videos

Other__________________________________________________

- **Did you want access to your biomarker testing report?**

No

Yes

**Section 3: Questions on demographic information**

- **What is your age in years?**
- **What is your sex?**

Female

Male

Intersex

Prefer not to answer

- **What is the highest level of education you have completed?**

No schooling completed

Elementary, primary or grade school

Secondary or high school

Attended some college and/or university

Graduated a 2-year college, technical school, and/or university

Graduated a 4-year college, technical school, and/or university

Post-graduate degree

Prefer not to answer
